# Interferon-Stimulated Genes that Target Retrovirus Translation

**DOI:** 10.3390/v16060933

**Published:** 2024-06-08

**Authors:** Niklas Jäger, Stefan Pöhlmann, Marina V. Rodnina, Shreya Ahana Ayyub

**Affiliations:** 1 Infection Biology Unit, German Primate Center—Leibniz Institute for Primate Research, 37077 Göttingen, Germany; njaeger@dpz.eu (N.J.); spoehlmann@dpz.eu (S.P.); 2 Faculty of Biology and Psychology, University Göttingen, 37073 Göttingen, Germany; 3 Max Planck Institute for Multidisciplinary Sciences, 37077 Göttingen, Germany; rodnina@mpinat.mpg.de

**Keywords:** HIV-1, retrovirus, interferon-stimulated genes, ZAP, Shiftless, PKR, Schlafen

## Abstract

The innate immune system, particularly the interferon (IFN) system, constitutes the initial line of defense against viral infections. IFN signaling induces the expression of interferon-stimulated genes (ISGs), and their products frequently restrict viral infection. Retroviruses like the human immunodeficiency viruses and the human T-lymphotropic viruses cause severe human diseases and are targeted by ISG-encoded proteins. Here, we discuss ISGs that inhibit the translation of retroviral mRNAs and thereby retrovirus propagation. The Schlafen proteins degrade cellular tRNAs and rRNAs needed for translation. Zinc Finger Antiviral Protein and RNA-activated protein kinase inhibit translation initiation factors, and Shiftless suppresses translation recoding essential for the expression of retroviral enzymes. We outline common mechanisms that underlie the antiviral activity of multifunctional ISGs and discuss potential antiretroviral therapeutic approaches based on the mode of action of these ISGs.

## 1. Introduction

The human immunodeficiency virus type 1 (HIV-1), the causative agent of AIDS, is the most prevalent human retrovirus, with 39 million infected individuals worldwide in 2022 (“https://www.who.int/data/gho/data/themes/hiv-aids” (accessed on 1 June 2024)). Also, infections caused by other human retroviruses like human T-lymphotropic virus-1 (HTLV-1) are associated with severe diseases [1,2,3]. The co-evolution of the human immune system and viral pathogens has led to the development of efficient and complex mechanisms to fight viral infection. The innate immune system represents the first line of defense against viral infection, and the interferon (IFN) system is an integral component of this defense. The IFN system is activated upon recognition of pathogen-associated molecular patterns (PAMPs) by pattern recognition receptors (PRRs), which triggers signaling cascades that commandeer the cell to produce IFN [4].

Type I IFNs (IFN-α, IFN-β, IFN-ε, IFN-κ, and IFN-ω in humans [5]) bind to the IFN-α receptor 1 (IFNAR1) and 2 (IFNAR2) heterodimers. The only representative of type II IFN, that is, IFN-γ, binds to the IFN-γ receptor (IFNGR) [6], while type III IFNs (IFN-λ1, IFN-λ2, IFN-λ3, and IFN-λ4 in humans [7,8,9]) engage the interleukin-10 receptor 2 (IL-10R2) and IFN-λ receptor 1 (IFNLR1) heterodimers. Receptor binding of IFNs initiates signaling through the Janus kinase signal transducer and activator of transcription (JAK-STAT) pathway, resulting in the upregulation of interferon-stimulated genes (ISGs), many of which encode proteins that restrict viral infection (termed restriction factors) [10]. Each IFN induces a specific set of ISGs, defined as the IFN signature [6], with type I IFNs inducing the expression of roughly 400 ISGs [11]. The IFN signature of type II IFN is associated with weaker antiviral effects as compared to that of type I IFN. However, it plays an important role in the expression of the major histocompatibility complex and activation of macrophages, inducing the inflammatory innate response required for clearance of infected cells [6,12,13]. The signatures associated with type I and type III IFN are roughly comparable and are mainly responsible for the antiviral effects of IFNs [14,15]. However, expression of ISGs induced by type I IFN is faster, more potent, and more short-lived as compared to ISGs induced by type III IFNs [9,15]. Notably, receptors for type I IFNs are ubiquitously expressed, while receptors for type III IFNs are predominantly found on epithelial cells and some immune cells [9,15]. Thus, one model suggests that type III IFN represents the first immune response to pathogens at the epithelial barriers, causing fewer inflammatory responses compared to type I IFN, which is subsequently activated when IFN III-mediated responses are insufficient to combat infection [15].

In the context of HIV-1 infection, an increase in IFNs and ISG expression is observed in the peripheral blood of infected individuals during the early phase of infection and is associated with a decline in viral load [16]. In contrast, during the chronic phase of HIV-1 infection, elevated levels of type I IFNs are associated with dysregulation and depletion of T cells, inflammation, and disease progression [17]. This phenotype can be reversed by blocking type I IFN signaling in HIV-1-infected humanized mice, which results in reduced HIV-1-induced inflammation, increased T cell numbers, and diminished HIV-1 reservoirs in the context of combination antiretroviral therapy (cART) [18,19]. Thus, IFN induction can limit HIV-1 replication in the early phase of infection, as also underlined by the marked anti-HIV-1 activity of recombinant IFN, but fails to control infection [10]. The antiviral activity of IFNs is associated with the expression of ISG-encoded restriction factors. HIV and other viruses have evolved elaborate mechanisms to evade or counteract these factors, reflecting an evolutionary arms race between host cell restriction factors and their viral antagonists (termed resistance factors) [10,20,21,22,23].

Restriction factors can inhibit one or more steps of the viral replication cycle, such as viral entry, viral replication, or viral budding [14]. Some inhibit viral infection directly by targeting viral components, such as the viral genome, proteins, or membrane, while others exert antiviral activity indirectly by modulating different steps of gene expression, including the translation of viral genes into proteins. In this review, we will discuss ISGs that inhibit retroviral infection by targeting mRNA translation, focusing on the proteins Schlafen 11 (SLFN11), SLFN12, SLFN13, Zinc Finger Antiviral Protein (ZAP), RNA-activated protein kinase (PKR), and Shiftless (SFL). Since retroviruses cause important diseases, understanding innate defenses against these viruses will provide important insights into viral pathogenesis and might define novel targets for intervention.

## 2. Translation of Retroviral Proteins as a Target for Restriction Factors

**Figure 1:**
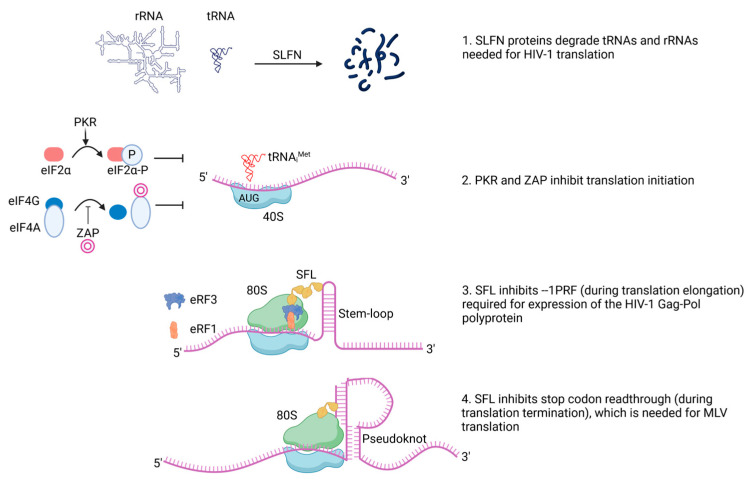
Interferon-stimulated genes (ISGs) that inhibit viral translation. (1) Schlafen (SLFN) proteins degrade certain cellular tRNAs and rRNAs, which, in turn, inhibit viral translation. (2) Zinc finger antiviral protein (ZAP) binds eIF4A and prevents eIF4G binding, which is required for the formation of the eIF4G complex and translation initiation. The RNA-activated protein kinase (PKR) phosphorylates eIF2α and inhibits translation initiation by preventing eIF2B from generating functional eIF2–GTP from eIF2–GDP. (3) Shiftless (SFL) inhibits the −1PRF required for HIV-1 translation, potentially by recruiting eRF3-eRF1 to the ribosome at the slippery site and causing premature translation termination. (4) SFL inhibits the stop codon readthrough required for MLV translation by an unknown mechanism. Created with BioRender.com.

Retroviruses exist in two forms: as cell-free (exogenous) infectious particles containing two copies of a single-stranded (ss) positive-sense (+) RNA genome and as cell-associated (endogenous) dsDNA elements, which are generated from viral RNA by the viral enzyme reverse transcriptase and integrated into the host genome by another viral enzyme, integrase [24]. Subsequently, cellular RNA polymerase II produces retroviral mRNAs, which are translated by host cell ribosomes [25]. Therefore, retroviral translation is most often inhibited by targeting host translation. The viral proteins Gag, Pol, and Env are encoded by all retroviruses and are essential for viral replication. Restriction factors interfere with the synthesis of these proteins in different ways (Figure 1). For example, the SLFN proteins target retroviral translation via alteration of the transfer RNA (tRNA) pool composition, which inhibits protein synthesis. The ISGs ZAP and PKR have a global inhibitory effect on cellular translation initiation, which also inhibits viral protein synthesis. In the context of HIV and murine leukemia virus (MLV) infection, Pol is produced as a Gag-Pol polyprotein, which depends on programmed −1 ribosomal frameshifting (−1PRF) (HIV) or programmed stop codon readthrough (MLV), processes that can be targeted by the restriction factor SFL (Figure 1). The ISGs discussed here also affect host translation and thereby host physiology [26,27,28,29], which is not discussed in the present review.

Translation is divided into the steps of initiation, elongation, termination, and ribosome recycling. Translation initiation, where the start codon (usually an AUG codon) and the open reading frame (ORF) are selected with the help of eukaryotic initiation factors (eIFs), is the most highly regulated step of protein synthesis [30,31]. Initiation begins with the recruitment of eIFs and a ternary complex of eIF2–GTP–Met-tRNA_i_^Met^ to the small ribosomal subunit, 40S. Binding of the majority of cellular mRNAs that contain a N^7^-methyl guanosine cap at their 5′ end requires the activity of the eIF4F protein complex (formed by eIF4A, eIF4E, and eIF4G). The bona fide start site is usually the first AUG codon from the 5′ end of the mRNA in an optimum nucleotide context. The start codon recognition triggers GTP hydrolysis by eIF2 and promotes remodeling of the complex, which facilitates the recruitment of the large ribosomal subunit, namely, 60S, to form the 80S ribosome that is ready to translate the selected ORF of the mRNA.

During elongation, the mRNA codons are read by tRNAs, which carry the amino acids into the catalytic center of the ribosome, where peptide bonds are made [32]. Each time an amino acid is incorporated into the growing peptide chain, the ribosome moves to the next codon, and these cycles of decoding, peptide bond formation, and translocation are repeated until the ribosome encounters a stop codon of the ORF. Translation terminates with the help of specialized proteins called release factors (RFs) [32]. eRF1 recognizes the stop codons and facilitates nascent polypeptide release upon GTP hydrolysis by eRF3. The release of the newly synthesized protein from the ribosome is followed by the recycling of ribosomes for a new round of translation. In general, protein synthesis is a highly regulated process, which provides a multitude of targets for retroviruses and host cells to modulate.

## 3. Schlafen Proteins Target Retroviral Translation via tRNA Degradation

The gene family encoding SLFN proteins was first described in the context of murine thymus development [33]. Over the years, *SLFN* genes were found to play a role in a variety of cellular functions, for instance, in the development and differentiation of T-cells [34], prevention of proliferation and invasion of cancer cells [35], sensitization of cancer cells to DNA-damaging drugs [36], and inhibition of viral replication [29,37,38,39,40,41,42,43]. The *SLFN* gene family is highly conserved across mammalian species [44,45,46,47]. In humans, six *SLFN* genes have been identified on chromosome 17. SLFN proteins contain a characteristic region called the “SLFN box”, which is located within the Schlafen core domain and contributes to antiviral activity [45,46,48,49]. The core domains of different SLFN proteins are highly conserved [48,50], except for the binding site for its substrate (RNA), which varies among the SLFN proteins and defines their substrate specificity and antiviral spectrum [51]. The human SLFN proteins SLFN5, SLFN11, SLFN13, and SLFN14 have an additional extended C-terminal domain harboring an RNA helicase-like motif [51,52,53,54]. Almost all *SLFN* genes are naturally expressed in monocytes, monocyte-derived dendritic cells, and T cells, and their expression is upregulated in response to IFN-α (SLFN5, SLFN11, SLFN12, and SLFN13) [55] and viral infection [42]. SLFN5 and SLFN11 have been detected in the nucleus [52,53,56], SLFN13 in the cytoplasm [51], and SLFN12 was found in both the nucleus and cytoplasm [54]. While SLFN5 blocks HIV transcription [56], SLFN11, SLFN12, and SLFN13 target HIV translation, as discussed below.

### 3.1. SLFN11 and SLFN12 Target Retroviral Translation via a Codon Usage Bias Mechanism

SLFN11 was first described as a restriction factor that interferes with the production of infectious HIV-1 (LAI strain) [39]. A comprehensive examination of the HIV replication cycle revealed that SLFN11 has no impact on reverse transcription, integration, transcription, nuclear export of viral RNA, viral budding, and release. Instead, SLFN11 interferes with the expression of viral proteins [39]. Structural and biochemical characterization of SLFN11, including in vitro cleavage assays and northern blot analysis of tRNAs, revealed that SLFN11 cleaves a number of tRNAs. This activity is mediated by the SLFN11 core domain, which exhibits a preference for tRNAs with a long variable loop, such as leucine- and serine-specific tRNAs. Thus, SLFN11 belongs to the group of tRNA endoribonucleases [49,57,58], and it is believed that reduction of cellular tRNA pools by SFLN11 interferes with translation of cellular and, predominantly, viral proteins. For instance, SLFN11 mediates the degradation of tRNA^Leu^, which reads the UUA codon (tRNA_UAA_^Leu^) [58]. Such tRNAs are more important for the expression of viral rather than cellular mRNAs because lentiviral genes, such as the HIV-1 gag-pol gene, possess a lower GC content relative to most host genes and thus tend to utilize synonymous codons that are rare in human genes [59,60,61]. HIV-1 compensates for its suboptimal codon usage by upregulating the expression of tRNAs that are underrepresented in the tRNA pool of lymphocytes. Degradation of HIV-1-utilized rare tRNAs by SLFN11 results in the preferential inhibition of viral protein synthesis [39,40,41,42]. Notably, the rare tRNA_UAA_^Leu^ recognizes 45% of all Leu codons in genes expressed late in HIV-1 infection, including *gag* and *pol* [62,63], highlighting the potential to inhibit HIV-1 protein translation in a codon usage-dependent manner. Adapting the codon usage of the HIV-1 gag gene to that of human genes rescues HIV-1 from inhibition by SLFN11, confirming that the anti-HIV activity of SLFN11 is based on discrimination of codon usage between host cells and viral transcripts [39].

In addition to codon bias-based inhibition of viral translation, SLFN11 can inhibit the translation of cellular proteins. For example, the human DNA damage response proteins ATR and ATM have comparable codon usage preferences as HIV-1, and their synthesis is inhibited by SFLN11, which enhances the sensitivity of tumor cells to chemotherapy [57,64]. Similarly, nonhuman primate SLFN11 inhibits host cell protein synthesis [29]. Finally, in line with SLFN11 targeting a translation mechanism essential for several viruses, SLFN11 inhibits retroviruses other than HIV, including murine stem cell virus (MSCV) [39], equine infectious anemia virus (EIAV) [40], prototype foamy virus (PFV) [41], and various other viruses with a pronounced codon bias, such as influenza A virus [42].

SLFN12 is believed to inhibit HIV via a mechanism similar to that employed by SLFN11 [65]. However, SFLN12 is less efficient at reducing tRNA levels and shows reduced anti-HIV activity as compared to SLFN11. One explanation might be that SLFN12 lacks the C-terminal helicase domain [44,47], as helicase-deficient mutants of SLFN11 are associated with reduced antiviral activity in the context of retroviral infection [41]. However, the nucleolytic activity is mainly linked to the SLFN core domain, and the role of the helicase domain in the context of tRNA cleavage remains to be demonstrated.

### 3.2. SLFN13 Targets Retroviral Translation via tRNA Degradation

SLFN13 also targets the translation of HIV mRNAs but employs a different mechanism than SLFN11 and SLFN12 [51]. Rather than targeting specific tRNAs, SLFN13 interferes with the translational machinery by degrading cytoplasmic rRNA and tRNAs regardless of their identity. In vitro cleavage assays demonstrated that SLFN13 is a tRNA/rRNA endoribonuclease that cleaves at the acceptor stem of mature cytoplasmatic tRNAs and digests 5S, 18S, and 28S rRNA, although cleavage sites remain to be defined [51]. SLFN13 mutants defective in tRNA cleavage are unable to inhibit HIV-1 infection, demonstrating that nucleolytic activity is essential for retroviral restriction [51]. SLFN13-mediated cleavage of cytoplasmic tRNA and rRNA triggers a global suppression of translation, thereby diminishing cell viability and inhibiting viral protein synthesis. An analogous mechanism has been described for the suicidal tRNase PrrC in *E. coli*, which is induced in response to bacteriophage T4 infection and cleaves the essential *E. coli* tRNA^Lys^, resulting in the death of the infected bacterium [51,66].

In conclusion, SLFN11, SLFN12, and SLFN13 interfere with the translation of host and viral mRNAs by cleaving essential components of the translational machinery [29,39,51,65]. Despite their functional redundancies, SLFN proteins differ in their substrate specificity for specific tRNAs and rRNA, their expression patterns, and/or cellular localization. The role of the cellular localization of SFLN proteins in the context of viral infection is poorly understood and remains to be elucidated to solve the interplay between different SLFN proteins during viral infection. Although current data largely suggest that the inhibitory effects of SLFN11, SLFN12, and SLFN13 are most likely due to their ability to interfere with the translation of host and viral mRNAs by cleaving translation-related RNAs, their direct impact on translation has not been measured. Conducting in vitro translation assays with recombinant SLFN proteins could help bridge this gap. Moreover, the correlation between tRNA abundance and SLFN expression needs to be elucidated in the context of HIV-infected individuals during different stages of viral infection.

## 4. ZAP Targets Retroviruses by Causing Viral RNA Decay and Inhibiting Translation Initiation

ZAP is part of the host’s arsenal of innate antiviral defenses against retroviruses and other RNA viruses [26]. Its ability to recognize CpG sequences in HIV-1 RNA and induce degradation contributes to the overall restriction of retroviral replication [26]. ZAP, also known as zinc finger CCCH-type containing, antiviral 1 (ZC3HAV1) or poly(ADP-ribose) polymerase (PARP) family member 13 (PARP13), stands out as a PRR that binds single-stranded RNA (ssRNA) and inhibits viral replication through various mechanisms, such as RNA degradation and translation inhibition [67].

Four ZAP isoforms have been identified, namely, ZAP-extralong (XL), ZAP-long (L), ZAP-medium (M), and ZAP-short (S), which are produced by alternative splicing [26]. ZAP-S and ZAP-M, but not ZAP-L and ZAP-XL, are upregulated by type I IFN. This indicates that IFNs can regulate not only transcription but also alternative splicing [26]. All isoforms share an N-terminal RNA-binding domain (RBD) with four CCCH-type zinc finger motifs (ZnF1–4), the central domain, including a fifth CCCH zinc finger motif (ZnF5), and two WWE (Trp-Trp-Glu) modules [26]. ZAP-L and ZAP-XL contain a C-terminal PARP-like domain, which lacks ADP-ribosyltransferase activity. ZAP-L and ZAP-S, which are the most abundant among ZAP proteins, have been extensively studied and are discussed in this review. An X-ray crystallography study of the ZAP RBD in complex with CpG dinucleotide-containing RNA showed that the highly basic second zinc finger motif has a pocket that selectively accommodates CpG dinucleotide bases [68]. ZAP-L and ZAP-S may function as homo- or heterodimers, forming oligomers on target RNA, where a single ZAP molecule only binds a single CpG [26]. The central domain binds poly(ADP-ribose), which is important for the full antiviral activity of these proteins, but the mechanism remains unclear [69]. The S-farnesylation of the PARP-like domain of ZAP-L and ZAP-XL promotes membrane localization by increasing protein hydrophobicity [70,71]. In contrast, ZAP-S, which lacks the PARP-like domain, is diffusely distributed in the cytoplasm [70]. ZAP-L and ZAP-S inhibit various retroviruses, including HIV-1, HTLV-1, avian leukosis virus (ALV), and MLV [26], via targeted RNA degradation and mRNA translation inhibition, and these two functions are likely linked.

In addition to CpG, other RNA sequences may also be recognized by ZAP. Notably, vertebrate genomes are CpG-poor, which allows ZAP to distinguish between viral and host genomes [72]. The position, structure, and context of CpG motifs influence ZAP’s antiviral efficacy, adding complexity to its selective targeting. For instance, CpG in the 5′ region of HIV-1 *env* is a better target for ZAP than in the 3′ end of the genome, and increasing the CpG frequency in the 5′ region of HIV-1 *env* has been linked to slower disease progression [73,74]. C(n7)GnCG has a high affinity for ZAP binding, where the additional C and G enhance the binding [75]. In retroviruses, ZAP has been shown to recruit a variety of RNAses for targeted mRNA decay [76]. ZAP directly interacts with the deadenylase PARN that shortens the poly(A) tail throughout 3′-5′ mRNA degradation. The decapping complex DCP1A-DCP2 and the exoribonuclease XRN1 interact with ZAP indirectly through the RNA helicase DDX17 to carry out 5′-3′ mRNA degradation.

In addition to targeting mRNA for degradation, ZAP-S expression inhibits translation of 5′ cap-containing HIV-derived mRNAs [77], and translation inhibition may augment the mRNA degradation effect. However, the two functions are independent because downregulation of PARN and Dcp2 in ZAP-expressing cells infected with an HIV-1 pseudovirus containing a luciferase reporter showed an expected decrease in degradation of HIV-derived mRNA but unchanged ZAP-mediated translation repression [77]. In this study, translation was specifically measured as luciferase activity normalized to pseudoviral mRNA levels. Thus, the translation levels were normalized for fluctuations in mRNA levels. ZAP-S interacts with eIF4A at its eIF4G-binding site, thus preventing the eIF4A-eIF4G interaction and inhibiting mRNA recruitment to the ribosome. It is important to note that ZAP-S does not inhibit translation of cellular GAPDH that does not contain ZAP-response elements (ZRE), which are viral RNA motifs that bind to ZAP. Therefore, it is likely that ZAP-S specifically inhibits the eIF4A-eIF4G interaction on ZRE-containing mRNAs. In contrast, a variant of the HIV pseudovirus with Hepatitis C virus (HCV) IRES-dependent translation initiation (which is independent of eIF4A-eIF4G interaction) is insensitive to both translation repression and mRNA decay by ZAP, indicating the importance of translation repression for mRNA decay. Generally, actively translating ribosomes protect mRNAs from decay by shielding them from RNases [78,79]. Repression of translation initiation results in a lack of protective ribosomes on the mRNA, leaving it vulnerable to ZAP-dependent RNA decay.

It is important to note that HIV proteins are synthesized by canonical cap-dependent translation during early infection [80]. However, the HIV-1 protease cleaves eIF4G and thereby largely incapacitates canonical translation initiation. As a consequence, HIV switches to internal ribosome entry site (IRES)-dependent translation initiation during late infection, which does not require the 5′ cap and eIF4F complex. Therefore, the HIV-1 translation inhibition function of ZAP is most effective during early infection.

Despite advancements in understanding ZAP’s antiviral activity, some challenges remain. For example, the optimal ZAP binding site and the full repertoire of ZAP cofactors are unknown. In summary, the past two decades of ZAP research have unraveled its intricate antiviral mechanisms, revealing a dynamic interplay between ZAP, viral RNA, and cellular factors. The multifunctionality of ZAP in recognizing, targeting, and restricting a diverse array of viruses underscores its important role in innate immunity.

## 5. PKR Targets Retroviral and Cellular Translation by Phosphorylating eIF2α

The type I IFN-induced RNA-activated protein kinase (PKR) is a 68 kDa protein with two dsRNA binding domains (dsRBD) at the N-terminus and a catalytic kinase domain at the C-terminus [81,82]. It exists in a monomeric, inactive form in cells but forms homodimers upon binding to dsRNA longer than 30 nucleotides. This leads to autophosphorylation of Thr446 and Thr451, which increases the catalytic activity of PKR [83]. Upon activation, PKR initiates the phosphorylation of multiple substrates, such as eIF2α. PKR phosphorylates eIF2α at Ser51. The phosphorylation inhibits the interaction of eIF2–GDP with its guanine nucleotide exchange factor, eIF2B, which is essential for initiating protein synthesis. Phosphorylation converts eIF2α from a substrate of eIF2B to a competitive inhibitor [84]. It has been shown with an HIV-1 infection model and subsequent immunoblots that HIV protein levels decline with increasing PKR phosphorylation but not with unphophorylated PKR [85]. The function of PKR is pivotal to both antiviral defense and cellular growth pathways [86].

HIV mRNAs contain a stem-loop structure called the trans-activation response (TAR) element at both termini. PKR acts as a dsRNA sensor during early HIV-1 infection, when low amounts of viral mRNAs are expressed, by binding to TAR, which transiently activates PKR [87]. HIV-1 employs various strategies to counteract PKR activation, including the Tat protein and cellular factors TRBP (TAR RNA-binding protein), ADAR1 (adenosine deaminase acting on RNA-1), and PACT (PKR activator) [27]. HIV-1 Tat protein has a sequence similarity to eIF2α; it binds to and sequesters PKR, thereby inhibiting the interaction of PKR with dsRNA [88,89]. TRBP binds to and sequesters dsRNA from PKR [90]. TRBP rescues HIV-1 replication in cells where viral expression was suppressed due to PKR [91]. In fact, cells with low TRBP expression, such as astrocytes, show higher PKR activation and lower HIV-1 replication [92]. ADAR1 directly binds to PKR and inhibits its activation [93]. PACT has two opposite roles, wherein it directly binds to and assists PKR activation under conditions of oxidative stress and at high expression levels of HIV-1, but inhibits PKR by forming a complex with TRBP and ADAR1 when levels of TRBP are high [85,94]. Interestingly, activated PKR also plays a role in splicing HIV-1 mRNA, indicating that HIV-1 has also co-opted PKR for its own benefit [95]. In summary, PKR acts as a restriction factor against HIV-1, and the virus employs various strategies to counteract its activation, involving viral and cellular factors.

## 6. Shiftless Targets mRNA Recoding Mechanisms

The ISG SFL was first described as an inhibitor of dengue virus (DENV) replication [96] and was subsequently shown to exert broad antiviral activity [28,97,98,99,100,101,102,103]. Human SFL is located on chromosome 19 (C19orf66) and encodes a 291 amino acid (aa)-long protein, which has a tendency to form oligomers [102]. According to AlphaFold predictions, the protein contains an α-helical N-terminal globular domain (residues 1–103), followed by the core domain (residues 104–240), a linker region (residues 241–260), and a C-terminal α-helix (residues 261–291). SFL expression is induced by all types of IFN (types I, II, and III) [96,97] and viral infection [104,105,106,107], indicating that SFL may play a role in various stress responses. Moreover, it can be upregulated in a multitude of cells, including monocytes and lymphocytes [97], which are targeted by several retroviruses [108,109,110].

In the context of retroviral infection, the ratio between the viral proteins Gag and Gag-Pol is important [111]. An imbalanced ratio of Gag and Gag-Pol disrupts viral replication, virion formation, and the infectivity of retroviruses such as HIV-1 or MLV [111,112,113,114,115]. Many retroviruses regulate their Gag to Gag-Pol stoichiometry by employing an mRNA recoding event, which enables the expression of Gag-Pol. Gag encodes the structural proteins matrix, capsid, nucleocapsid, and p6, whereas Pol encodes the viral enzymes protease, reverse transcriptase, and integrase. Immature Gag and Gag-Pol are incorporated into viral particles during viral assembly, and the viral protease, a component of the Pol portion of the Gag-Pol polyprotein, mediates the proteolytic processing of both Gag and Gag-Pol, which converts immature particles into infectious virions. SFL inhibits −1PRF [28] and stop codon readthrough [116], which are required for the expression of Gag-Pol and consequently for the formation of infectious retroviral particles [28]. The following sections discuss how SFL interferes with recoding of viral mRNAs.

### 6.1. Shiftless Targets Programmed −1 Ribosomal Frameshifting

During frameshifting, the translating ribosome shifts its position in the ORF by one or two nucleotides backwards (−PRF) or forwards (+PRF) relative to the reading frame selected at the initiation stage. This usually occurs when the ribosome encounters the so-called frameshifting stimulation element (FSE) of the mRNA, consisting of the slippery sequence and a secondary structure element, such as a stem-loop structure or a pseudoknot. After slippage, the ribosome continues translation in a new reading frame [117,118]. Many viruses, including HIV-1, utilize −1PRF to increase the coding capacity of their genome and to regulate relative gene expression [119,120,121,122,123]. The FSE for HIV-1 −1PRF is located at the border of the *gag* and *pol* mRNA sequences and comprises two cis-acting RNA elements, the heptameric slippery sequence U UUU UUA, followed by a downstream stem-loop. The secondary structure acts as a stimulatory element for the slippery sequence, which facilitates ribosomal slippage.

In addition to HIV-1, SFL inhibits −1PRF in a variety of retroviruses, such as Rous sarcoma virus (RSV), HTLV-2, mouse mammary tumor virus (MMTV), HIV-2, and simian immunodeficiency virus (SIV) [28]. Using dual-luciferase assays, the frameshift efficiency of these retroviruses and their inhibition by SFL were demonstrated by inserting the viral FSE between renilla luciferase (Rluc) and firefly luciferase (Fluc), with Fluc in the −1 reading frame. In this system, translation of Fluc occurred only in the case of −1PRF, with the ratio of Fluc to Rluc indicating frameshifting efficiency [28]. In agreement with SFL-mediated suppression of −1PRF in these reporter systems, IFN-α reduces the −1PRF-dependent translation of Gag-Pol in HIV-1-infected MT4 cells, whereas the knockdown of SFL partially rescues HIV-1 from this inhibitory effect [28]. SFL also targets the −1PRF signal of the host PEG10 RNA, indicating that SFL can inhibit −1PRF-dependent translation of both viral and cellular mRNAs [28], but the significance of the latter is unclear.

SFL blocks −1PRF by interacting with actively translating ribosomes through the ribosomal proteins uL5 and eS31, the ribosome release factor eRF3, and the stem-loop element of the FSE, ultimately resulting in premature translation termination at the FSE [28]. Premature translation termination was examined using a reporter construct that carried the −1PRF signal of HIV-1 flanked by an upstream GST with an N-terminal Flag-tag and a downstream Fluc. However, the premature translation termination product in the context of authentic HIV-1 Gag-Pol remains to be detected. The current hypothesis is that during −1PRF, the prolonged pausing of the ribosome at the FSE [124,125,126] may bring uL5 into close proximity to eS31, which might provide a unique arrangement enabling simultaneous binding of SFL to both the ribosome and the FSE, thereby blocking the ribosome in a nonproductive state [28]. Since SFL multimerizes, it can simultaneously interact with proteins and mRNAs that are not located in close proximity [28,102]. A splice variant of SFL, known as Shiftless short (SFLS), lacks amino acid residues 164–199 and is unable to interact with the FSE, form multimers, or inhibit −1PRF [28,102], but can be utilized as a tool to study SFL functions. For instance, while SFL can self-interact, SFLS cannot, and SFL can interact with SFLS [102]. This indicates that the 36 aa region missing in SFLS interacts with another region of SFL. In this context, it has also been demonstrated that the propensity of SFL to multimerize correlates with its ability to bind to the ribosome [103], emphasizing the role of SFL-SFL interactions during ribosome stalling at the FSE. However, the specific regions within SFL that are responsible for these interactions remain to be elucidated.

In addition to preventing −1PRF, SFL causes premature translation termination, resulting in the production of a truncated 0-frame product [28]. eRF1 in complex with eRF3 promotes the rescue and release of stalled ribosomes [127]. SFL binds to eRF3, which might facilitate the rescue [28]. Knockdown of eRF3 diminishes the production of the truncated 0-frame product but has no impact on the expression of the −1PRF product. This suggests that the interaction with the ribosome rescue machinery and the inhibition of −1PRF represent two independent modes of action of SFL. The relative contributions of these distinct modes of action of SFL to the restriction of HIV-1 infection remain to be elucidated.

Mutational studies of the zinc-binding motif in SFL suggest that this region plays an important role in SFL binding to HIV-1 mRNA and SFL-mediated inhibition of −1PRF [103]. This study also revealed that inhibition of −1PRF and inhibition of HIV-1 infection are separable traits of SFL. One could speculate that the capacity of SFL to restrict HIV-1 in an −1PRF-independent manner relies on promoting the ribosome rescue process, which leads to the accumulation of truncated 0-frame products and thereby disruption of protein stoichiometry. However, other modes of action are possible, including interference with the expression of other viral proteins, such as Env. Furthermore, a recent study has shown that both SFL overexpression and depletion can reduce the abundance of reporter mRNAs, independent of whether they possess an FSE [128]. Although it is important to note that this study was carried out with frameshift reporter systems rather than with authentic viruses [128], this finding may suggest that SFL plays a role in modulating RNA stability and might be involved in cellular RNA decay pathways, as described in the context of other viral infections [97,99].

### 6.2. Shiftless Targets Programmed Stop Codon Readthrough

SFL inhibits not only −1PRF but also another translational recoding mechanism, known as programmed stop codon readthrough, which enables the ribosome to pass through the termination codon to continue elongation [103,116]. Similar to −1PRF, stop codon readthrough is mainly used by viruses to expand the coding capacity of their genome and to regulate relative gene expression required for efficient viral replication [129,130]. Upon translational readthrough, a tRNA misreads the stop codon, resulting in continued elongation of the polypeptide chain until the next stop codon is found. Normally, this occurs very infrequently. The spontaneous readthrough frequency for human RNA sequences is usually rather low, in the range of 0.001% per stop codon, but can be enhanced by several factors, including the codon context [131].

MLV relies on programmed stop codon readthrough for Gag-Pol expression [132,133]. SFL co-expression reduces Gag-Pol but not Gag levels in transfected cells and diminishes the formation of infectious MLV particles [103]. In agreement with these findings, inhibition of MLV readthrough by SFL was also demonstrated in an in vitro translation assay using rabbit reticulocyte lysate (RRL) extracts and a linearized MLV readthrough reporter construct [116]. In contrast, another study reported only a minor effect of SFL on MLV infectivity [28], but this discrepancy could be due to lower SFL expression levels attained in that study. Finally, it remains to be directly demonstrated that SFL-mediated inhibition of MLV is due to the inhibition of programmed stop codon readthrough.

Programmed −1PRF and stop codon readthrough exhibit commonalities that could offer insights into how SFL inhibits programmed stop codon readthrough. The frequency of HIV-1 −1PRF and MLV stop codon readthrough depends on secondary structure elements in the RNA, for example, pseudoknot (MLV) or stem loop (HIV-1) structures, and it is tempting to speculate that SFL changes the conformational plasticity of these RNA elements, which impedes RNA unfolding during translation and affects ribosome interactions with these structures [134]. Moreover, as SFL interacts with eRF3 in the context of HIV-1 infection [28], it may stabilize the eRF1–eRF3 binding to the ribosome, thereby potentially impeding erroneous decoding of the stop codon by a tRNA. While recent data indicate this interesting role for SFL, the detailed mechanism of how SFL interferes with stop codon readthrough remains to be elucidated.

## 7. Conclusions and Future Perspectives

The arms race between ISGs and viruses has driven the emergence of multifaceted mechanisms of viral inhibition, and individual ISGs can exert several antiviral activities. SFL is a prime example since the protein not only inhibits retroviruses via at least two mechanisms (this review) but is also active against various other viruses [135]. For example, in the context of Japanese Encephalitis Virus (JEV) infection, SFL inhibits −1PRF and targets the viral NS3 protein for lysosomal degradation [101]. To inhibit Kaposi’s sarcoma-associated herpesvirus (KSHV), SFL triggers the disassembly of stress granules and P bodies, which alters the fate of RNAs with regards to translational arrest or degradation [99]. In the case of the Dengue virus (DENV), SFL binds to the viral 3′ UTR, the La-related protein 1 (LARP1), and the poly(A)-binding protein cytoplasmic 1 (PABPC1), and thereby restricts the translation of viral RNA [96]. Since LARP1 and PABPC1 are known to play a role in RNA decay as components of stress granules and P bodies, it is possible that SFL plays a role in DENV RNA degradation [96]. In the context of Hepatitis C virus (HCV) infection, SFL prevents the formation of intracellular replication organelles, termed the membranous web, and localizes to stress granules [98]. Since the studies discussed above indicate that SFL frequently localizes to stress granules in the context of infection, it would be interesting to determine whether stress granule localization also contributes to SFL-mediated inhibition of retrovirus infection. ZAP isoforms also inhibit multiple viruses, but unlike SFL, its principle strategy is to target viral RNAs for degradation [26]. Interestingly, ZAP-S does reduce SARS-CoV-2 −1PRF by inhibiting the folding of the pseudoknot of the FSE, but is inactive against HIV −1PRF [136]. SLFN11 and SLFN12 have been implicated in the maintenance of HIV latency, and increased expression of SLFN12 in peripheral blood mononuclear cells (PBMCs) of HIV-infected patients was associated with reduced levels of intracellular HIV-1 RNA [65]. Thus, selective upregulation of SLFN12 and/or SLFN11 in PBMCs may support infection control. Another therapeutic approach includes the shock and kill strategy, which relies on a two-step process [137]. The first step is focused on the reactivation of latent HIV, which could be achieved by the knockout of SLFN proteins, such as SLFN 12. In the second step, reactivated HIV can then be targeted and eradicated by the host immune system or antiretroviral drugs.

Leveraging ISGs for therapeutic purposes has been explored in various contexts, particularly in the treatment of viral infections. Recent research has uncovered a novel role for ISGs, including PKR and ZAP-S, demonstrating that they can activate the innate immune response against viral infection, thereby strengthening this response [138]. For example, ZAP-S can activate the RIG-1 pathway, which, in turn, activates a type I IFN response, and PKR is known to regulate IFN-β. However, a more detailed analysis of the activated host pathways and the triggered antiviral effectors is required before the therapeutic potential of such ISGs can be harnessed. Another promising antiviral therapeutic strategy would be to inhibit viral resistance mechanisms against ISGs. For example, inhibition of HIV-1 Tat would not only inhibit viral transcription but also lead to a higher population of activated PKR (Section 5). In fact, it would be interesting to study the roles of known Tat inhibitors, such as triptolide, in PKR activation [139]. In contrast, no such retroviral resistance factors have been found against SFL, SLFN proteins, and ZAP isoforms. Applications of ZAP research in synthetic virology, such as synonymous genome recoding to increase the CpG content of HIV-1, also hold promise for live attenuated vaccine development but require further research [140]. A greater understanding of the structure-function relationship of the ISGs will also pave the way for the design of antiviral agents such as peptide mimetics of functionally relevant regions of these ISGs [141].

It is important to study ISG antiviral activity and its biological relevance at physiological expression levels and in appropriate cell and animal models instead of overexpression studies. ZAP knockout in cultured primary mouse embryonic fibroblasts results in increased MLV replication, confirming that this protein can restrict retroviruses under physiological conditions [142]. However, such data are lacking for the other ISGs discussed here. Although a prominent antiviral effect of SFL has been demonstrated in the context of Zika virus infection using SFL knockout mice [143], comparable data are missing for HIV and other retroviruses. This holds true for other ISGs such as ZAP, PKR, and SLFN proteins as well, where comprehensive retroviral studies in knockout mouse models are lacking. Notably, elevated levels of SLFN11 were detected in HIV-1-infected CD4^+^ T-cells from elite controllers, who maintain undetectable viral load in the absence of antiretroviral therapy [144], suggesting that SLFN11 might contribute to viral inhibition under these conditions. Overall, a complete understanding of the regulation of viral protein synthesis and the ISGs that inhibit it is required to develop effective antiviral agents.
